# Population-based nationwide incidence of complications after gastrectomy for gastric adenocarcinoma in Finland

**DOI:** 10.1093/bjsopen/zrad101

**Published:** 2023-10-21

**Authors:** Emilia Putila, Olli Helminen, Mika Helmiö, Heikki Huhta, Aapo Jalkanen, Raija Kallio, Vesa Koivukangas, Arto Kokkola, Simo Laine, Elina Lietzen, Johanna Louhimo, Sanna Meriläinen, Vesa-Matti Pohjanen, Tuomo Rantanen, Ari Ristimäki, Jari V Räsänen, Juha Saarnio, Eero Sihvo, Vesa Toikkanen, Tuula Tyrväinen, Antti Valtola, Joonas H Kauppila

**Affiliations:** Surgery Research Unit, Medical Research Center Oulu, Oulu University Hospital and University of Oulu, Oulu, Finland; Surgery Research Unit, Medical Research Center Oulu, Oulu University Hospital and University of Oulu, Oulu, Finland; Division of Digestive Surgery and Urology, Turku University Hospital, Turku, Finland; Surgery Research Unit, Medical Research Center Oulu, Oulu University Hospital and University of Oulu, Oulu, Finland; Department of Surgery, University of Helsinki and Helsinki University Hospital, Helsinki, Finland; Department of Oncology and Radiotherapy, Oulu University Hospital, Oulu, Finland; Surgery Research Unit, Medical Research Center Oulu, Oulu University Hospital and University of Oulu, Oulu, Finland; Department of Surgery, University of Helsinki and Helsinki University Hospital, Helsinki, Finland; Division of Digestive Surgery and Urology, Turku University Hospital, Turku, Finland; Division of Digestive Surgery and Urology, Turku University Hospital, Turku, Finland; Department of Surgery, University of Helsinki and Helsinki University Hospital, Helsinki, Finland; Surgery Research Unit, Medical Research Center Oulu, Oulu University Hospital and University of Oulu, Oulu, Finland; Cancer and Translational Medicine Research Unit, Medical Research Center Oulu, University of Oulu and Oulu University Hospital, Oulu, Finland; Department of Surgery, University of Eastern Finland and Kuopio University Hospital, Kuopio, Finland; Department of Pathology, HUSLAB, HUS Diagnostic Center, Helsinki University Hospital and University of Helsinki, Helsinki, Finland; Applied Tumour Genomics Research Program, Research Programs Unit, Faculty of Medicine, University of Helsinki, Helsinki, Finland; Department of General Thoracic and Oesophageal Surgery, Heart and Lung Centre, University of Helsinki and Helsinki University Hospital, Helsinki, Finland; Surgery Research Unit, Medical Research Center Oulu, Oulu University Hospital and University of Oulu, Oulu, Finland; Department of Surgery, Central Finland Central Hospital, Jyväskylä, Finland; Department of Cardiothoracic Surgery, Heart Center, Tampere University Hospital and University of Tampere, Tampere, Finland; Department of Gastroenterology and Alimentary Tract Surgery, Tampere University Hospital, Tampere, Finland; Department of Surgery, University of Eastern Finland and Kuopio University Hospital, Kuopio, Finland; Surgery Research Unit, Medical Research Center Oulu, Oulu University Hospital and University of Oulu, Oulu, Finland; Department of Molecular Medicine and Surgery, Karolinska Institutet and Karolinska University Stockholm, Stockholm, Stockholm, Sweden

## Abstract

**Background:**

The incidence of postoperative complications after gastrectomy for gastric cancer is not well known. More population-based studies using established complication classifications are needed for international comparison. The aim of this study was to evaluate the population-based incidence of postoperative complications after gastrectomy for gastric cancer.

**Methods:**

This population-based study based on the Finnish National Esophago-Gastric Cancer Cohort included all patients at least 18 years of age undergoing gastrectomy for gastric adenocarcinoma in Finland during 2005–2016. The occurrence of complications 30 and 90 days after surgery was graded based on the Esophagectomy Complications Consensus Group definitions and the severity of complications was assessed using the Clavien–Dindo scale.

**Results:**

This study included a total of 2196 patients. Postoperative complications occurred in 906 (41.3 per cent) of patients during 30 days after surgery and in 946 (43.1 per cent) during 90 days after surgery. Clavien–Dindo grade III or higher complications occurred in 375 (17.1 per cent) of patients. The most common complications 90 days after surgery by Esophagectomy Complications Consensus Group upper-level categories were gastrointestinal (*n* = 438; 19.9 per cent), including anastomotic leak, infectious (*n* = 377; 17.2 per cent) and pulmonary (*n* = 335; 15.3 per cent) complications. Postoperative mortality rate was occurred in 72 (3.3 per cent) patients within 30 days and in 161 (7.3 per cent) patients within 90 days after surgery. The median duration of postoperative hospital stay was 9 days (interquartile range 4–14).

**Conclusions:**

Postoperative complications are common across all types of gastrectomy and the majority occur during the first 30 postoperative days. This study informs the patients and caregivers of the expected outcomes of gastrectomy.

## Teaser

This population-based nationwide cohort study included 2196 patients who underwent surgery for gastric adenocarcinoma and describes the incidence of complications after gastrectomy. The incidence of 30-day complications after gastrectomy was 41.3 per cent and the incidence of 90-day complications was 43.1 per cent. The most common 90‐day complications were gastrointestinal (19.9 per cent), infectious (17.2 per cent) and pulmonary (15.3 per cent).

## Introduction

Gastric cancer is the third leading cause of cancer death worldwide^1^. The standard treatment for local or locally advanced gastric cancer is gastrectomy, which is associated with high mortality and morbidity rates, a long hospital stay and a high reoperation rate^2^. Population-based studies on postoperative complications of gastric cancer surgery from Western countries are sparse. In a previous Dutch study, the incidence of postoperative complications after gastrectomy was 43 per cent, while the 30-day mortality rate was 4.4 per cent and readmission occurred in 14 per cent of the patients^2^. A Japanese nationwide study of surgically resected gastric cancer resulted in a 30-day mortality rate of 0.5 per cent and 90-day mortality rate of 1.7 per cent after surgery^3^.

The severity of complications is commonly graded using the Clavien–Dindo classification for surgical complications based on the type of treatment needed^4^, but without differentiating between the types of complications. Previous nationwide analyses have been reported using the Esophagectomy Complications Consensus Group (ECCG) standardized list of complications^5^, providing a comparison point for a national analysis.

The aim of the present study was to describe the population-based nationwide incidence of complications after gastrectomy for gastric adenocarcinoma in Finland according to the Clavien–Dindo and the ECCG classifications, grouped by the type of surgery and surgical approach.

## Methods

### Study design

This study was a population-based nationwide retrospective cohort study in Finland during 2005–2016, using the Finnish National Esophago-Gastric Cancer Cohort (FINEGO)^6^. All patients who underwent gastrectomy for gastric adenocarcinoma in Finland were included in this study. Patients who underwent another type of surgery other than gastrectomy (for example palliative gastric bypass), those with other histology than gastric adenocarcinoma or without a histological confirmation of cancer were excluded, as well as those without available data.

### Data collection

All potentially eligible patients were identified from the Finnish cancer and patient registries^7,8^. Records of patients with gastric cancer or tumour diagnosis in the Finnish Cancer Registry or the Finnish Patient Registry and a relevant surgical code in the Patient Registry were retrieved from the respective hospitals and healthcare units and screened for eligibility by expert surgeons^9^.

The patient registry provided data on date of surgery, age, sex and co-morbidity. Cancer stage information was updated according to TNM 8th edition^10^. Following the ascertainment of eligibility, patient records, including surgical charts and pathology assessments, were evaluated by expert upper gastrointestinal surgeons, and information on tumour and treatment characteristics, as well as complications, was retrieved and input to the common database using the REDCap (Research Electronic Data Capture) web-based tool hosted at the University of Oulu, Finland^11,12^. Clavien–Dindo grade I complications were not collected, as the assessment of these complications was deemed unfeasible given the retrospective design, the low clinical relevance and subjectivity. Statistics Finland provided the reliable and 100 per cent complete mortality rate data^13^.

### Outcomes

The primary outcome was occurrence of any postoperative complication during 30 and 90 days. Secondary outcomes were types of 30-day and 90-day complications grouped by the ECCG upper-level categories, the severity of the complications using the Clavien–Dindo classification^4^, reoperation rate, length of intensive care unit (ICU) stay, duration of hospital stay and in-hospital, 30-day or 90-day mortality rates.

Clavien–Dindo grades IIIa and higher were considered to be major complications. The consensus by the ECCG was used to separately evaluate each complication, and to group them in upper-level complication categories (pulmonary, cardiac, gastrointestinal, urologic, thromboembolic, neurological, infectious, wound and other)^5^ as shown in *Table S1*. Reoperation rate was defined as surgical interventions performed in the operating theatre, including both with and without general anaesthesia.

### Statistical methods

Statistical analysis was descriptive. The patient and tumour characteristics, complications, reoperation rate, length of postoperative ICU stay, duration of hospital stay, and mortality rate are presented as frequencies and percentages. Complications are also reported for total and distal gastrectomy, open and laparoscopic surgery, as well as curative and palliative gastrectomy. IBM SPSS version 27 (Armonk, NY, USA) was used for data management and analysis.

## Results

### Patients

**Fig. 1:**
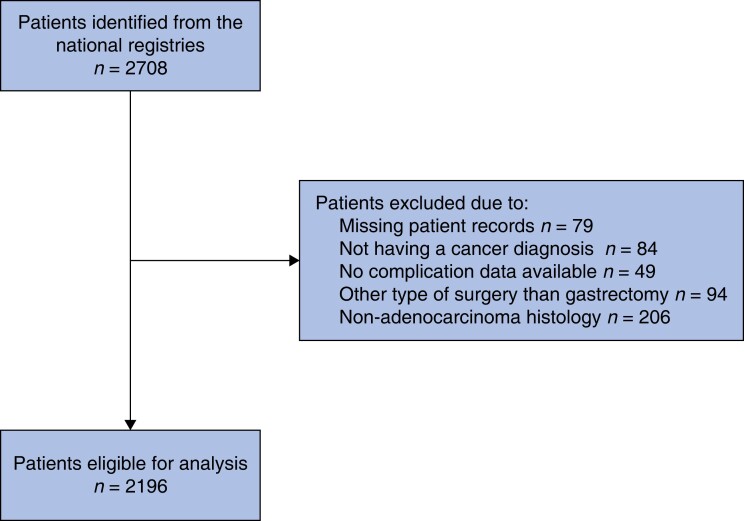
Exclusion criteria of patients eligible for analysis

From 2005 to 2016, a total of 2708 patients were identified from the national registries and assessed for eligibility; of those, a total of 2196 patients were included in the analysis (*Fig. 1*). The majority were males (*n* = 1227; 55.9 per cent) with a median age of 71 years at the time of surgery, with no co-morbidity (*n* = 1104; 50.3 per cent), and with a pathological stage III (*n* = 761; 34.7 per cent) cancer (*Table 1*). Most patients (*n* = 1326; 60.4 per cent) underwent a total gastrectomy. Open surgery (*n* = 2093; 93.4 per cent) was the most common surgery type. The preoperative intent of surgery was mainly curative (*n* = 2003; 91.2 per cent).

**Table 1:** Characteristics of patients undergoing gastrectomy for gastric adenocarcinoma during 2005–2016 in Finland

	*n* (%)
**Total**	2196 (100)
**Time interval**	
2005–2008	867 (39.5)
2009–2012	720 (32.8)
2013–2016	609 (27.7)
**Age at surgery (years)**	
Median	71
i.q.r.	(55–87)
**Sex**	
Male Female	1227 (55.9) 969 (44.1)
**Co-morbidity**	
0	1104 (50.3)
1	663 (30.2)
2	263 (12.0)
3 or more	166 (7.6)
**pTNM/ypTNM**	
0	11 (0.5)
I	523 (23.8)
II	631 (28.7)
III	761 (34.7)
IV	229 (10.4)
missing	41 (1.9)
**Histology**	
Adenocarcinoma	2196 (100)
**Preoperative surgery intent**	
Curative	2003 (91.2)
Palliative	178 (8.1)
Rescue after definitive chemoradiotherapy	2 (0.1)
Unclear	15 (0.6)
**Surgery type**	
Total gastrectomy	1326 (60.4)
Distal gastrectomy	840 (38.3)
Proximal gastrectomy	24 (1.1)
Wedge resection	6 (0.3)
**Surgical approach**	
Open surgery	2093 (95.3)
Laparoscopic surgery	103 (4.7)
**Preoperative neoadjuvant therapy**	
Yes	302 (13.8)
No	1887 (85.9)
Missing	7 (0.3)

i.q.r., interquartile range.

Postoperative complication rates 30 and 90 days after surgery according to the ECCG upper- and lower-level categories are reported in *Table 2*.

**Table 2:** Postoperative complications after gastrectomies for gastric cancer during 2005–2016 in Finland according to ECCG Annals of Surgery 2015^5^

Complications	*n* (%)
Total	2196
30-day complications	906 (41.3)
90-day complications	946 (43.1)
Major complications	375 (17.1)
**Clavien–Dindo classification**	
No complications or grade I	1256 (57.2)
Grade II	565 (25.7)
Grade III	218 (9.9)
Grade IV	92 (4.2)
Grade V*	65 (3.0)
**ECCG categories 30-day complications**	
Pulmonary	327 (14.9)
Pneumonia	254 (11.6)
Pleura effusion requiring additional drainage procedure	102 (4.6)
Pneumothorax requiring treatment	5 (0.2)
Atelectasis mucous plugging requiring bronchoscopy	7 (0.3)
Respiratory failure requiring reintubation	38 (1.7)
Acute aspiration	19 (0.9)
Acute respiratory distress syndrome	15 (0.7)
Chest tube maintenance for air leak for >10 d after surgery	0 (0.0)
Cardiac	147 (6.7)
Cardiac arrest requiring CPR	18 (0.8)
Myocardial infarction	32 (1.5)
Dysrhythmia atrial requiring treatment	66 (3.0)
Dysrhythmia ventricular requiring treatment	4 (0.2)
Congestive heart failure requiring treatment	62 (2.8)
Pericarditis requiring treatment	1 (0.0)
Gastrointestinal	354 (16.1)
Oesophagoenteric leak from anastomosis, staple line or localized Conduit necrosis	105 (4.8)
Conduit necrosis/failure	0 (0.0)
Ileus defined as small bowel dysfunction preventing or delaying enteral feeding	98 (4.5)
Small bowel obstruction	13 (0.6)
Feeding J-tube complication	8 (0.4)
Pyloromyotomy/pyloroplasty complication	0 (0.0)
*Clostridium difficile* infection	15 (0.7)
Gastrointestinal bleeding requiring intervention or transfusion	83 (3.8)
Delayed conduit emptying requiring intervention or delaying discharge or requiring maintenance of NG drainage >7 d after surgery	35 (1.6)
Pancreatitis	8 (0.4)
Liver dysfunction	22 (1.0)
Urologic	96 (4.4)
Acute renal insufficiency (defined as doubling of baseline creatinine)	26 (1.2)
Acute renal failure requiring dialysis	8 (0.4)
Urinary tract infection	47 (2.1)
Urinary retention requiring reinsertion of urinary catheter, delaying discharge or discharge with urinary catheter	24 (1.1)
Thromboembolic	36 (1.6)
Deep venous thrombosis	7 (0.3)
Pulmonary embolus	22 (1.0)
Stroke	8 (0.4)
Peripheral thrombophlebitis	0 (0.0)
Neurologic	54 (2.5)
Recurrent nerve injury	1 (0.0)
Other neurologic injury	16 (0.7
Acute delirium	38 (1.7)
Delirium tremens	0 (0.0)
Infectious	356 (16.2)
Wound infection requiring opening wound or antibiotics	57 (2.6)
Central i.v. line infection requiring removal or antibiotics	12 (0.5)
Intrathoracic/intra-abdominal abscess	163 (7.4)
Generalized sepsis	53 (2.4)
Other infections requiring antibiotics	124 (5.6)
Wound	42 (1.9)
Wound dehiscence	40 (1.8)
Acute abdominal wall dehiscence/hernia	2 (0.1)
Acute diaphragmatic hernia	0 (0.0)
Other	44 (2.0)
Chyle leak	16 (0.7)
Reoperation on for reasons other than bleeding, anastomotic leak or conduit necrosis	13 (0.6)
Multiple organ dysfunction syndrome	17 (0.8)
**ECCG categories 90-day complications**	
Pulmonary	335 (15.3)
Pneumonia	258 (11.7)
Pleura effusion requiring additional drainage procedure	106 (4.8)
Pneumothorax requiring treatment	6 (0.3)
Atelectasis mucous plugging requiring bronchoscopy	7 (0.3)
Respiratory failure requiring reintubation	38 (1.7)
Acute aspiration	19 (0.9)
Acute respiratory distress syndrome	15 (0.7)
Chest tube maintenance for air leak for >10 d after surgery	0 (0.0)
Cardiac	156 (7.1)
Cardiac arrest requiring CPR	22 (1.0)
Myocardial infarction	34 (1.5)
Dysrhythmia atrial requiring treatment	69 (3.1)
Dysrhythmia ventricular requiring treatment	4 (0.2)
Congestive heart failure requiring treatment	67 (3.1)
Pericarditis requiring treatment	1 (0.0)
Gastrointestinal	438 (19.9)
Oesophagoenteric leak from anastomosis, staple line or localized Conduit necrosis	108 (4.9)
Conduit necrosis/failure	0 (0.0)
Ileus defined as small bowel dysfunction preventing or delaying enteral feeding	107 (4.9)
Small bowel obstruction	22 (1.0)
Feeding J-tube complication	10 (0.5)
Pyloromyotomy/pyloroplasty complication	0 (0.0)
*Clostridium difficile* infection	16 (0.7)
Gastrointestinal bleeding requiring intervention or transfusion	85 (3.9)
Delayed conduit emptying requiring intervention or delaying discharge or requiring maintenance of NG drainage >7 d after surgery	38 (1.7)
Pancreatitis	10 (0.5)
Liver dysfunction	24 (1.1)
Urologic	100 (4.6)
Acute renal insufficiency (defined as doubling of baseline creatinine)	28 (1.3)
Acute renal failure requiring dialysis	8 (0.4)
Urinary tract infection	49 (2.2)
Urinary retention requiring reinsertion of urinary catheter, delaying discharge or discharge with urinary catheter	24 (1.1)
Thromboembolic	46 (2.1)
Deep venous thrombosis	8 (0.4)
Pulmonary embolus	30 (1.4)
Stroke	10 (0.5)
Peripheral thrombophlebitis	0 (0.0)
Neurologic	54 (2.5)
Recurrent nerve injury	1 (0.0)
Other neurologic injury	17 (0.8)
Acute delirium	38 (1.7)
Delirium tremens	0 (0.0)
Infectious	377 (17.2)
Wound infection requiring opening wound or antibiotics	61 (2.8)
Central i.v. line infection requiring removal or antibiotics	12 (0.5)
Intrathoracic/intra-abdominal abscess	176 (8.0)
Generalized sepsis	56 (2.6)
Other infections requiring antibiotics	135 (6.1)
Wound	45 (2.0)
Wound dehiscence	42 (1.9)
Acute abdominal wall dehiscence/hernia	2 (0.1)
Acute diaphragmatic hernia	1 (0.0)
Other	50 (2.3)
Chyle leak	17 (0.7)
Reoperation on for reasons other than bleeding, anastomotic leak or conduit necrosis	17 (0.8)
Multiple organ dysfunction syndrome	18 (0.8)
90-day reoperation on	181 (8.2)
Hospital stay, days (i.q.r.)	9 (4–14)
ICU stay, days (i.q.r.)	0 (0–0)
30-day mortality rate	72 (3.3)
90-day mortality rate	161 (7.3)

CPR, cardiopulmonary resuscitation; ECCG, Esophagectomy Complications Consensus Group; ICU, intensive care unit; i.q.r., interquartile range; NG, naso-gastric tube. *In-hospital mortality rate.

Postoperative complication rates according to surgical approach, resection type and curative *versus* palliative-intent gastrectomy are reported in *Tables 3*, *4*, and *5* respectively.

**Table 3:** **Postoperative complications after open and laparoscopic gastrectomy for gastric cancer during 2005–2016 in Finland according to ECCG Annals of Surgery 2015** ^5^

Complications	Open gastrectomy	Laparoscopic gastrectomy
	*n* (%)	*n* (%)
Total	2093	103
30-day complications	865 (41.3)	41 (39.8)
90-day complications	903 (43.1)	43 (41.7)
Major complications	359 (17.5)	16 (15.7)
**Clavien–Dindo**		
No complications or grade I	1196 (57.1)	60 (58.3)
Grade II	538 (25.7)	27 (26.6)
Grade III	206 (9.8)	12 (11.7)
Grade IV	89 (4.3)	3 (2.9)
Grade V*	64 (3.1)	1 (1.0)
**ECCG 30-day complications**		
Pulmonary	313 (15.0)	14 (13.6)
Cardiac	141 (6.7)	6 (5.8)
Gastrointestinal	343 (16.4)	11 (10.7)
Urologic	90 (4.3)	6 (5.8)
Thromboembolic	35 (1.7)	1 (1.0)
Neurologic	53 (2.5)	1 (1.0)
Infectious	342 (16.3)	14 (13.6)
Wound	40 (1.9)	2 (1.9)
Other	43 (2.1)	1 (1.0)
**ECCG 90-day complications**		
Pulmonary	321 (15.3)	14 (13.6)
Cardiac	150 (7.2)	6 (5.8)
Gastrointestinal	423 (20.2)	15 (14.6)
Urologic	94 (4.5)	6 (5.8)
Thromboembolic	45 (2.2)	1 (1.0)
Neurologic	53 (2.5)	1 (1.0)
Infectious	361 (17.2)	16 (15.5)
Wound	43 (2.1)	2 (1.9)
Other	48 (2.3)	2 (1.9)
Duration of hospital stay, days (i.q.r.)	9 (4–14)	8 (5–11)
ICU stay, days (i.q.r.)	0 (0–0)	0 (0–0)
30-day mortality rate	69 (3.3)	3 (2.9)
90-day mortality rate	158 (7.5)	3 (2.9)

ECCG, Esophagectomy Complications Consensus Group; ICU, intensive care unit; i.q.r., interquartile range. *In-hospital mortality rate.

**Table 4:** **Postoperative complications after total and distal gastrectomy for gastric cancer during 2005–2016 according to ECCG Annals of Surgery 2015** ^5^

Complications	Total gastrectomy	Distal gastrectomy
	*n* (%)	*n* (%)
Total	1326	840
30-day complications	549 (41.4)	342 (40.7)
90-day complications	578 (43.6)	352 (41.9)
Major complications	239 (18.0)	127 (15.1)
**Clavien–Dindo**		
No complications or grade I	750 (56.6)	491 (58.5)
Grade II	337 (25.4)	222 (26.4)
Grade III	138 (10.4)	78 (9.3)
Grade IV	61 (4.6)	28 (3.3)
Grade V*	40 (3.0)	21 (2.5)
**ECCG 30-day complications**		
Pulmonary	213 (16.1)	105 (12.5)
Cardiac	84 (6.3)	59 (7.0)
Gastrointestinal	218 (16.4)	129 (15.4)
Urologic	52 (3.9)	41 (4.9)
Thromboembolic	22 (1.7)	13 (1.5)
Neurologic	24 (1.8)	28 (3.3)
Infectious	231 (17.4)	123 (14.6)
Wound	27 (2.0)	15 (1.8)
Other	32 (2.4)	9 (1.1)
**ECCG 90-day complications**		
Pulmonary	220 (16.6)	106 (12.6)
Cardiac	87 (6.6)	65 (7.7)
Gastrointestinal	265 (20.0)	165 (19.6)
Urologic	54 (4.1)	43 (5.1)
Thromboembolic	27 (2.0)	18 (2.1)
Neurologic	24 (1.8)	28 (3.3)
Infectious	246 (18.6)	129 (15.4)
Wound	28 (2.1)	17 (2.0)
Other	38 (2.9)	9 (1.1)
Duration of hospital stay, days (i.q.r.)	10 (5–15)	9 (4–14)
ICU stay, days (i.q.r.)	0 (0–0)	0 (0–0)
30-day mortality rate	38 (2.9)	30 (3.6)
90-day mortality rate	93 (7.0)	64 (7.6)

ECCG, Esophagectomy Complications Consensus Group; ICU, intensive care unit; i.q.r., interquartile range. *In-hospital mortality.

**Table 5:** **Postoperative complications after curative-intent and palliative-intent gastrectomy for gastric cancer during 2005–2016 in Finland according to ECCG Annals of Surgery 2015** ^5^

Complications	Curative-intent gastrectomy	Palliative-intent gastrectomy
	*n* (%)	*n* (%)
Total	2003	178
30-day complications	818 (40.8)	81 (45.5)
90-day complications	856 (42.7)	82 (46.1)
Major complications	336 (16.8)	34 (19.1)
**Clavien–Dindo**		
No complications or grade I	1152 (57.5)	97 (54.5)
Grade II	515 (25.7)	47 (26.4)
Grade III	200 (10.0)	16 (9.0)
Grade IV	85 (4.2)	6 (3.4)
Grade V*	51 (2.5)	12 (6.7)
**ECCG 30-day complications**		
Pulmonary	303 (15.1)	23 (12.9)
Cardiac	130 (6.5)	14 (7.9)
Gastrointestinal	319 (15.9)	33 (18.5)
Urologic	89 (4.4)	6 (3.4)
Thromboembolic	29 (1.4)	6 (3.4)
Neurologic	47 (2.3)	6 (3.4)
Infectious	327 (16.3)	28 (15.7)
Wound	34 (1.7)	6 (3.4)
Other	39 (1.9)	4 (2.2)
**ECCG 90-day complications**		
Pulmonary	310 (15.5)	24 (13.5)
Cardiac	137 (6.8)	16 (9.0)
Gastrointestinal	396 (19.8)	39 (21.9)
Urologic	92 (4.6)	7 (3.9)
Thromboembolic	38 (1.9)	7 (3.9)
Neurologic	47 (2.3)	6 (3.4)
Infectious	343 (17.1)	32 (18.0)
Wound	37 (1.8)	6 (3.4)
Other	45 (2.2)	4 (2.2)
Duration of hospital stay, days (i.q.r.)	9 (4–14)	8 (5–11)
ICU stay, days (i.q.r.)	0 (0–0)	0 (0–0)
30-day mortality rate	58 (2.9)	11 (6.2)
90-day mortality rate	118 (5.9)	40 (22.5)

ECCG, Esophagectomy Complications Consensus Group; ICU, intensive care unit; i.q.r., interquartile range. *In-hospital mortality rate.

### 30-day morbidity rate

Some 906 (41.3 per cent) patients had a postoperative complication at 30 days. The most common ECCG upper-level categories were infectious (*n* = 356; 16.2 per cent), gastrointestinal (*n* = 354; 16.1 per cent) and pulmonary (*n* = 327; 14.9 per cent) complications (*Table 2*). The most common complications were pneumonia (*n* = 254; 11.6 per cent), followed by intra-abdominal abscess (*n* = 163; 7.1 per cent) and other infections not specified under other complications (*n* = 124; 5.6 per cent). The 30-day mortality rate was 3.3 per cent (*n* = 72) as shown in *Table 2*.

### 90-day morbidity rate

Some 946 (43.1 per cent) patients had 90-day complications. The most common ECCG upper-level categories were gastrointestinal (*n* = 438; 19.9 per cent), infectious (*n* = 377; 17.2 per cent) and pulmonary (*n* = 335; 15.3 per cent) complications. The most common complications were pneumonia (*n* = 258; 11.7 per cent), intrathoracic or intra-abdominal abscess (*n* = 176; 8.0 per cent) and other infections requiring antibiotics (*n* = 135; 6.1 per cent). Clavien–Dindo grade ≥III complications occurred in 375 (17.1 per cent) patients. Reoperation was required in 181 (8.2 per cent) patients. The median length of ICU stay was 0 days (interquartile range (i.q.r.) 0–0), and the median duration of hospital stay was 9 days (i.q.r. 4–14). Total in-hospital and 90-day mortality rates were 3.0 per cent (*n* = 65) and 7.3 per cent (*n* = 161) respectively (*Table 2*).

## Discussion

This Finnish population-based nationwide study presents the incidence of 30- and 90-day morbidity rate after gastrectomy, as well as those of laparoscopic- and open-, total- and distal- as well as curative-intended and palliative gastrectomy. The most common categories of complications were gastrointestinal, followed by infectious, pulmonary and thromboembolic complications.

The 2019 Gastrectomy Complications Consensus Group (GCCG)^14^ classified major specific complications after gastrectomy, and it could be argued that it is the preferable method to report complications. The GCCG list was published when the FINEGO data collection was on-going, and items between the GCCG and ECCG lists were relatively similar, so the ECCG list could also be considered a valid classification method.

A recent population-based national study on the occurrence of complications after gastrectomy for gastric cancer using the ECCG classification was based on the Dutch DUCA database (study number population = 928)^2^ and it found a complication rate of 43 per cent. The 30-day complication rate in the present study was 41.3 per cent. The occurrence of major complications was quite similar (19 per cent *versus* 17.1 per cent). The most common complications grouped by the ECCG in the Dutch study were gastrointestinal (18 per cent), pulmonary (17 per cent) and infectious (9 per cent), while in this study they were infectious (16.2 per cent), gastrointestinal (16.1 per cent) and pulmonary (14.9 per cent) complications. The infectious complication rate was higher in the present study and could be due to the early administration of antibiotic therapy in the past without a clear infectious focus. A European retrospective observational study (*n* = 1349) from high-volume hospitals using the GCCG classification estimated the overall incidence of complications, 90 days after surgery, at 29.8 per cent, the most common complications being non-surgical infections (23 per cent), anastomotic leak (9.8 per cent) and other postoperative abnormal fluid from drainage and/or abdominal collections (9.3 per cent)^15^. The lower incidence of complications in that study could be explained by the stricter criteria in the GCCG classification. Thirty-day mortality rates were similar (3.3 per cent *versus* 3.6 per cent) between the studies, but 90-day mortality rates were slightly higher in the present study (7.3 per cent *versus* 4.3 per cent), due to the inclusion of patients who underwent a palliative gastrectomy.

A Dutch population-based study found fewer wound complications and a shorter duration of hospital stay after laparoscopic compared with open gastrectomy^16^. Laparoscopic distal gastrectomy was also associated with lower overall and wound complications compared with open gastrectomy in a Korean study^17^. In the present study, results were relatively similar, but there was a lower 90-day mortality rate in the laparoscopic gastrectomy group. Patients who underwent laparoscopic surgery had less advanced disease due to the learning curve of laparoscopic gastrectomy in many centres.

For total and distal gastrectomy, a population-based Dutch study found that the most common complications after total or subtotal gastric cancer surgery were pulmonary (15 per cent), anastomotic leakage (7 per cent) and cardiac (6 per cent) complications^18^. In the present study the incidence of complications was similar between total and distal gastrectomy. Observational studies have suggested better survival with palliative gastrectomy in advanced gastric cancer compared with conservative treatment^19,20^, but this hypothesis was not supported by the results of the Japanese randomized REGATTA trial^21^. In the present study, similar 90-day complications after palliative- and curative-intended gastrectomy were observed, but there were longer durations of hospital stay, more major complications, and a much higher 90-day mortality rate after palliative- *versus* curative-intended gastrectomy, probably due to the weaker state of health of these patients.

Occurrence of a complication could impair health-related quality of life of patients in the long term^22^. As a population-based nationwide study, this study informed the caregivers and patients of the incidence of complications after gastrectomy for gastric cancer. Furthermore, the incidence of complications with typical surgical approaches, surgery types and curative and palliative intent were described, facilitating patient–caregiver discussions, and providing realistic information in specific settings and circumstances.

The strengths of this study include the inclusion of a large population, allowing accurate estimates and reducing selection bias, and the ascertainment of complications by expert surgeons improved the quality of data.

Limitations were due to its retrospective nature, and the possible lack of data that could not be retrieved. Some categories, such as laparoscopic and palliative surgery groups, had a relatively small number of patients, but the distributions of complications were relatively similar across different groups. Nevertheless, strict conclusions from the subcategories of gastric cancer surgery should be avoided. The proportion of patients receiving preoperative neoadjuvant therapy was only 14.8 per cent, which may limit the generalizability of the results in these patients.

In conclusion, this population-based, nationwide cohort study suggests that complications occur commonly after gastrectomy for gastric adenocarcinoma and across surgical approaches, surgery types and intents. The majority of complications occur during the first 30 days and fewer are observed between 30 and 90 days after surgery.

## Supplementary Material

zrad101_Supplementary_Data

## Data Availability

The data can be shared for research purposes upon request by contacting the Chief Investigator, J.K., but may be restricted by and require complimentary permissions from the ethical committee and relevant original data holders.
