# Supplementary material for: Population-based nationwide incidence of complications after gastrectomy for gastric adenocarcinoma in Finland
Source: BJS Open. 2023 Oct 21;7(5):zrad101. doi: 10.1093/bjsopen/zrad101 (PMC10590062; doi:10.1093/bjsopen/zrad101)
Supplement: zrad101_Supplementary_Data [file zrad101_supplementary_data.docx]

**Population-based nationwide incidence of complications after gastrectomy for gastric adenocarcinoma in Finland.**

Authors: Emilia Putila, BM^1^, Olli Helminen, MD, PhD^1^, Mika Helmiö, MD, PhD^2^, Heikki Huhta, MD, PhD^1^, Aapo Jalkanen, MD^3^, Raija Kallio, MD, PhD^4^, Vesa Koivukangas, MD, PhD^1^, Arto Kokkola, MD, PhD^3^, Simo Laine, MD, PhD^2^, Elina Lietzen, MD, PhD^2^, Johanna Louhimo, MD, PhD^3^, Sanna Meriläinen, MD, PhD^1^, Vesa-Matti Pohjanen, MD, PhD^5^, Tuomo Rantanen, MD, PhD^6^, Ari Ristimäki, MD, PhD^7,8^, Jari V. Räsänen, MD, PhD^9^, Juha Saarnio, MD, PhD^1^, Eero Sihvo, MD, PhD^10^, Vesa Toikkanen, MD, PhD^11^, Tuula Tyrväinen, MD, PhD^12^, Antti Valtola, MD^6^, Joonas H. Kauppila^1,13^, on behalf of the FINEGO group

Affiliations:

^1^Surgery Research Unit, Medical Research Center Oulu, Oulu. University Hospital and University of Oulu, Oulu, Finland

^2^Division of Digestive Surgery and Urology, Turku University Hospital, Turku, Finland

^3^Department of Surgery, University of Helsinki and Helsinki University Hospital, Helsinki, Finland

^4^Department of Oncology and Radiotherapy, Oulu University Hospital, Oulu, Finland

^5^Cancer and Translational Medicine Research Unit, Medical Research Center Oulu, University of Oulu and Oulu University Hospital, Oulu, Finland

^6^Department of Surgery, University of Eastern Finland and Kuopio University Hospital, Kuopio, Finland

^7^Department of Pathology, HUSLAB, HUS Diagnostic Center, Helsinki University Hospital and University of Helsinki, Helsinki, Finland

^8^Applied Tumour Genomics Research Program, Research Programs Unit, Faculty of Medicine, University of Helsinki, Helsinki, Finland

^9^Department of General Thoracic and Oesophageal Surgery, Heart and Lung Centre, University of Helsinki and Helsinki University Hospital, Helsinki, Finland

^10^Department of Surgery, Central Finland Central Hospital, Jyväskylä, Finland

^11^Department of Cardiothoracic Surgery, Heart Center, Tampere University Hospital and University of Tampere, Tampere, Finland

^12^Department of Gastroenterology and Alimentary Tract Surgery, Tampere University Hospital, Tampere, Finland

^13^Department of Molecular Medicine and Surgery, Karolinska Institutet and Karolinska University Stockholm, Sweden

**Corresponding author** Emilia Putila, Department of Surgery, Aapistie 5, PO. Box 5000, 90014 University of Oulu, Oulu Finland

**ORCID ID**: 0000-0001-6135-189X

**Supplementary Materials - Index**

| **Supplementary Figures and Tables** |  |
| --- | --- |
| Supplementary table 1 | *pag. 3-4* |
| **References** | *pag. 5* |
|  |  |

**Supplementary Figures and Tables**

Supplementary Table 1: Complications and their groupings, as defined by the Esophagectomy Complications Consensus Group (ECCG)^2^.

| Pulmonary |
| --- |
| Pneumonia |
| Pleura effusion requiring additional drainage procedure |
| Pneumothorax requiring treatment |
| Atelectasis mucous plugging requiring bronchoscopy |
| Respiratory failure requiring reintubation |
| Acute aspiration |
| Acute respiratory distress syndrome |
| Chest tube maintenance for air leak for > 10 d postoperatively |
| Cardiac |
| Cardiac arrest requiring CPR |
| Myocardial infarction |
| Dysrhythmia atrial requiring treatment |
| Dysrhythmia ventricular requiring treatment |
| Congestive heart failure requiring treatment |
| Pericarditis requiring treatment |
| Gastrointestinal |
| Esophagoenteric leak from anastomosis, staple line or localized Conduit necrosis |
| Conduit necrosis/failure |
| Ileus defined as small bowel dysfunction preventing or delaying enteral feeding |
| Small bowel obstruction |
| Feeding J-tube complication |
| Pyloromyotomy/pyloroplasty complication |
| *Clostridium difficile* infection |
| Gastrointestinal bleeding requiring intervention or transfusion |
| Delayed conduit emptying requiring intervention or delaying discharge or requiring maintenance of NG drainage >7d postoperatively |
| Pancreatitis |
| Liver dysfunction |
| Urologic |
| Acute renal insufficiency (defined as doubling of baseline creatinine) |
| Acute renal failure requiring dialysis |
| Urinary tract infection |
| Urinary retention requiring reinsertion of urinary catheter, delaying discharge, or discharge with urinary catheter |
| Thromboembolic |
| Deep venous thrombosis |
| Pulmonary embolus |
| Stroke (CVA) |
| Peripheral thrombophlebitis |
| Neurologic |
| Recurrent nerve injury |
| Other neurologic injury |
| Acute delirium |
| Delirium tremens |
| Infectious |
| Wound infection requiring opening wound or antibiotics |
| Central IV line infection requiring removal or antibiotics |
| Intrathoracic/intra-abdominal abscess |
| Generalized sepsis |
| Other infections requiring antibiotics |
| Wound |
| Wound dehiscence |
| Acute abdominal wall dehiscence/hernia |
| Acute diaphragmatic hernia |
| Other |
| Chyle leak |
| Reoperation for reasons other than bleeding, anastomotic leak or conduit necrosis |
| Multiple organ dysfunction syndrome |

**References**

^2^van der Werf LR, Busweiler LAD, van Sandick JW, van Berge Henegouwen MI, Wijnhoven BPL; Dutch Upper GI Cancer Audit (DUCA) group. Reporting National Outcomes After Esophagectomy and Gastrectomy According to the Esophageal Complications Consensus Group (ECCG). Ann Surg. 2020 Jun;271(6):1095-110.
